# Supplementary material for: Genomic Dissection of a Wild Region in a Superior Solanum pennellii Introgression Sub-Line with High Ascorbic Acid Accumulation in Tomato Fruit
Source: Genes (Basel). 2020 Jul 24;11(8):847. doi: 10.3390/genes11080847 (PMC7466095; doi:10.3390/genes11080847)
Supplement: Supplementary file 1 [file genes-11-00847-s001.zip › Table S3.docx]

**Supplementary Table S3:** Number of reads of R182 and M82 before and after the quality check (QC). Number of unique, multi- and unmapped reads, the mismatch ratio as well as the number of reads assigned to genic features (Assigned-GTF) are reported.

| **Sample** | **Raw reads** | **Reads after QC** | **Unique** | **Multi-mapped** | **Unmapped** | **Mismatch-ratio** | | **Assigned-GTF** |  |  |  |
| --- | --- | --- | --- | --- | --- | --- | --- | --- | --- | --- | --- |
| **M82_A_BR** | 15903732 | 13074281 | 12117888 (92.6849%) | 138852 (1.06202%) | 817541 (6.25305%) | 0.13% | 11433102 (87.4473%) | | | |  |
| **M82_A_MR** | 19339109 | 15932768 | 15000480 (94.1486%) | 179336 (1.12558%) | 752952 (4.72581%) | 0.14% | 14133108 (88.7047%) | | | |  |
| **M82_B_BR** | 16611207 | 13546582 | 12795928 (94.4587%) | 139429 (1.02926%) | 611225 (4.51202%) | 0.14% | 12042153 (88.8944%) | | | |  |
| **M82_B_MR** | 15100453 | 12238345 | 11557624 (94.4378%) | 124189 (1.01475%) | 556532 (4.54744%) | 0.13% | 10905638 (89.1104%) | | | |  |
| **M82_C_BR** | 17800125 | 14621763 | 13619219 (93.1435%) | 129355 (0.884674%) | 873189 (5.97184%) | 0.14% | 12831375 (87.7553%) | | | |  |
| **R182_A_BR** | 18543040 | 15314862 | 13977563 (91.268%) | 219354 (1.43229%) | 1117945 (7.29974%) | 0.16% | 12909440 (84.2935%) | | | |  |
| **R182_A_MR** | 17594548 | 14319971 | 13701082 (95.6781%) | 162976 (1.1381%) | 455913 (3.18376%) | 0.14% | 12769430 (89.1722%) | | | |  |
| **R182_B_BR** | 21331796 | 17655616 | 16564023 (93.8173%) | 185457 (1.05041%) | 906136 (5.13228%) | 0.15% | 15483911 (87.6996%) | | | |  |
| **R182_B_MR** | 15489939 | 12869277 | 12185580 (94.6874%) | 116896 (0.908334%) | 566801 (4.4043%) | 0.13% | 11387731 (88.4877%) | | | |  |
| **R182_C_BR** | 20060622 | 16505640 | 15074792 (91.3312%) | 221745 (1.34345%) | 1209103 (7.32539%) | 0.19% | 13980601  (84.702%) | | | |  |
| **R182_C_MR** | 15249274 | 12650062 | 11992054 (94.7984%) | 138759 (1.0969%) | 519249 (4.10472%) | 0.15% | 11303299 (89.3537%) | | | |  |
