# Supplementary material for: Genomic Dissection of a Wild Region in a Superior Solanum pennellii Introgression Sub-Line with High Ascorbic Acid Accumulation in Tomato Fruit
Source: Genes (Basel). 2020 Jul 24;11(8):847. doi: 10.3390/genes11080847 (PMC7466095; doi:10.3390/genes11080847)

**Supplementary Figure 1.** Distribution of the variants observed comparing the RNA-Seq reads from the R182 sub-line and S. lycopersicum (M82) across the 12 tomato chromosomes. Red rectangle highlights the peak of variants corresponding to the wild introgression on chromosome 7

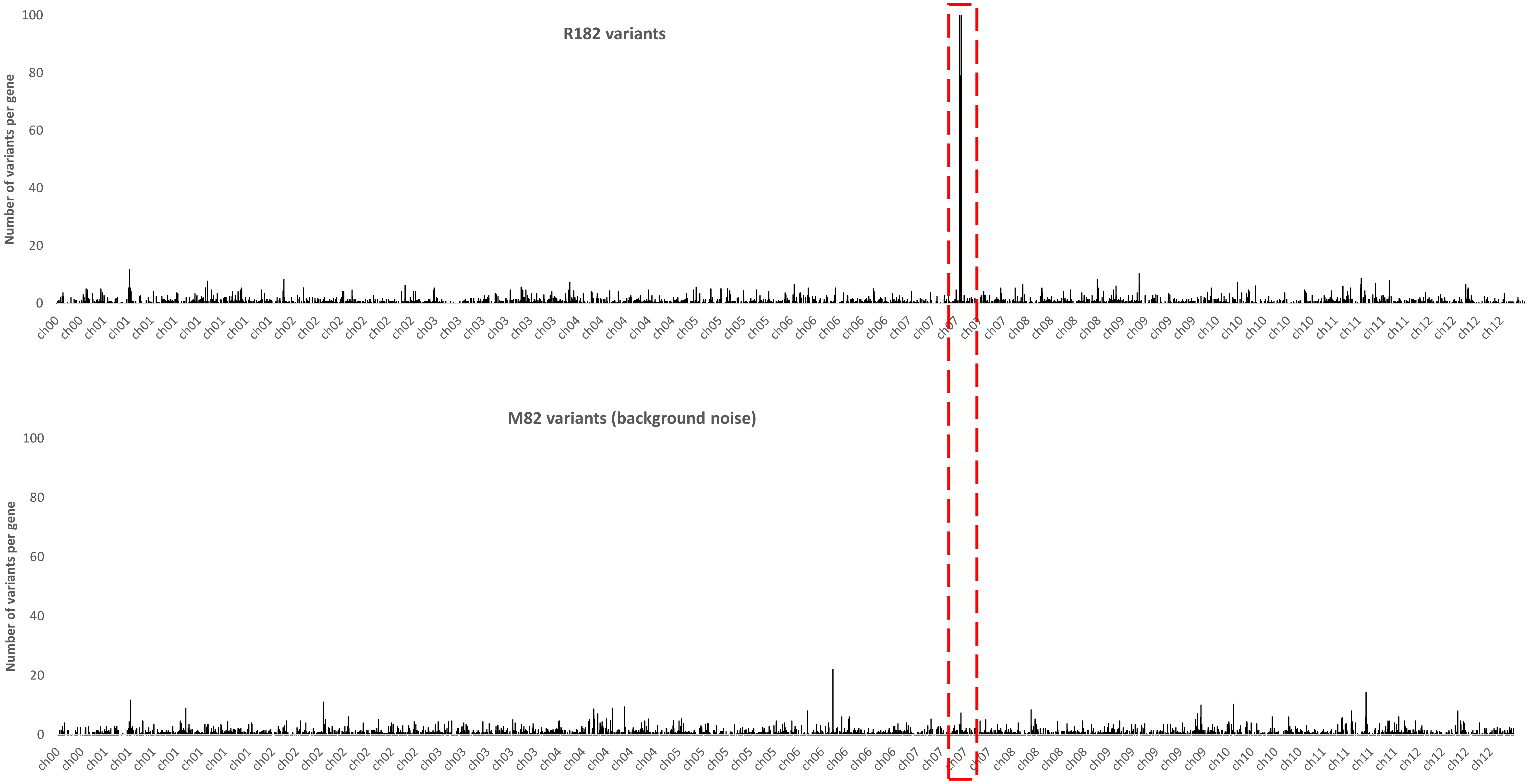

Supplement: Supplementary file 1 [file genes-11-00847-s001.zip › Figure S1.pdf]
